# Residents and staff perceptions of a pediatric clinical teaching unit in a large tertiary care center in Saudi Arabia

**DOI:** 10.1186/s12909-022-03155-7

**Published:** 2022-02-08

**Authors:** Tahir Kamal Hameed, Syed Furrukh Jamil, Hamad Abdullah Alkhalaf

**Affiliations:** 1 King Saud Bin Abdulaziz University for Health Sciences, Riyadh, Saudi Arabia; 2 Department of Pediatrics, King Abdullah Specialized Children’s Hospital, King Abdulaziz Medical City – Riyadh, Ministry of National Guard - Health Affairs, PO Box 22490, Riyadh, 11426 Saudi Arabia; 3 King Abdullah International Medical Research Center, Ministry of National Guard – Health Affairs, Riyadh, Saudi Arabia

**Keywords:** Pediatrics, Graduate medical education, Teaching

## Abstract

**Background:**

The clinical teaching unit (CTU) is a commonly used model of patient care and teaching. Despite being a common model of care, very few studies have looked at its impact on the education of trainees. In addition, it is a relatively new structure for pediatric inpatient care in Saudi Arabia. The purpose of this study was to compare postgraduate trainees (residents) and staff perceptions of the old and the new (the CTU) inpatient team structures, and to evaluate the impact of the CTU on residents’ education.

**Methods:**

An online survey was sent to nurses, pediatric residents, and attending physicians who worked under both structures. Questions for residents were adopted from the National training survey of the General Medical Council, United Kingdom.

**Results:**

A total of 147 pediatric healthcare workers completed the survey (97 nurses, 39 residents, and 11 attending physicians), most of whom worked in both the old and new inpatient team structures. More than 97% of residents reported being supervised by their attending on a daily basis in the CTU structure as compared with 15% in the old structure. A higher proportion of residents favored the old structure in terms of the opportunity it provided to develop their leadership skills. Eighty-seven percent of nurses believed the CTU had improved patient safety of pediatric inpatients. Overall, 82% of residents, 91% of nurses, and 100% of attending physicians favoured the CTU structure over the old inpatient model.

**Conclusions:**

Our study shows that pediatric residents and staff perceived the CTU structure as superior to the old inpatient team structure, especially in terms of patient safety. Although the CTU seemed to have a positive impact on residents’ education, this must be further examined especially with respect to its impact on residents’ leadership skills.

**Supplementary Information:**

The online version contains supplementary material available at 10.1186/s12909-022-03155-7.

## Background

The clinical teaching unit (CTU) first emerged in medical education in Canada approximately 50 years ago [1]. The CTU was originally defined by Evans et al. as a “Clinical Teaching Unit, Division or Service, which may be an entire hospital or designated hospital area, is one providing undergraduate and graduate medical education, not limited to the intern year, under the auspices of a Faculty of Medicine of a Canadian university…medical care is the function of the team of staff physician, resident, intern and clinical clerk, based on the principle of graded responsibility commensurate with competence and level of training” [2]. In other countries the concept of teaching ward rounds has been applied for a long time; in fact the first teaching ward round in the UK was introduced in 1660 [3].

In January 2016, the Department of Pediatrics in King Abdullah Specialized Children’s Hospital (KASCH) changed the inpatient general pediatric service to the CTU structure. The old inpatient structure consisted of four general pediatric teams with one team on call each day. Each team consisted of a senior resident, junior residents and interns with several consultants (attendings) assigned to each team. In addition, each day there was a different attending on call. Thus pediatric residents, who do 4-week rotations in general pediatric wards, had patients under multiple attendings at the same time. The contact time of residents with attendings was mainly during “post-admission rounds” on new patients, which made it difficult for attendings to give detailed feedback to trainees. In addition, front line staff such as nurses had to liaise with multiple attendings for the day-to-day patient care. Considering these concerns, and the goal to improve patient care and safety, the general pediatric inpatient service was restructured into the CTU. The key changes made with the shift to the CTU were attendings being assigned for 1 to 2 weeks of service with daily rounds and admissions being divided equally between the four CTU teams. The CTU team consisted of an attending physician (consultant), a senior resident, junior residents and interns. Medical students were also assigned to the CTU team based on their rotation schedules. The CTU team would make daily rounds on all patients (new and old).

A study by Szecket et al. on an inpatient internal medicine service showed that restructuring the service by splitting admissions between the teams improved the discharge rates and shortened their median length of hospital stay [4]. Another study assessed many interventions in a residency teaching service. One intervention was to maximize the number of patients a team shared with a single attending to improve team efficiency and patient-based teaching. The interventions resulted in improved resident and student experience [5].

Despite the CTU being implemented in institutions worldwide, only few studies have investigated its impact on the medical education of postgraduate trainees (residents). Moreover, the CTU is a relatively new concept in pediatrics in the Kingdom of Saudi Arabia. With the implementation of the CTU in our hospital, we aimed to compare residents and staff perceptions of the old and new (the CTU) inpatient structures, and to evaluate the impact of the CTU structure on trainees’ education. We hypothesized that the new structure would provide better education and supervision of pediatric residents while improving the communication among members of the multidisciplinary team including nursing staff.

## Methods

### Study design

A questionnaire was developed for residents based on the National training survey of the General Medical Council in the UK [6]. The questionnaire was administered to residents in 2017 and SurveyMonkey was used to collect the responses online. The study was triangulated by collecting shorter survey questionnaires from staff (nurses and attending physicians). The study was conducted in KASCH. Only residents, nurses, and attending physicians who worked in both the old and new inpatient team structure (the CTU) were surveyed. Before starting our study, we obtained ethical approval from the Institutional Review Board of King Abdullah International Medical Research Center, Ministry of National Guard – Health Affairs, Riyadh.

### Data analysis and statistics

Descriptive statistics (frequencies and percentages) were used to summarize the data. In addition, qualitative data were collected to obtain more detailed perceptions about the old and new inpatient team structures. The old and new inpatient team structures were compared using the chi-square test, and the analysis was performed using the R V3.6 software.

## Results

**Fig. 1:**
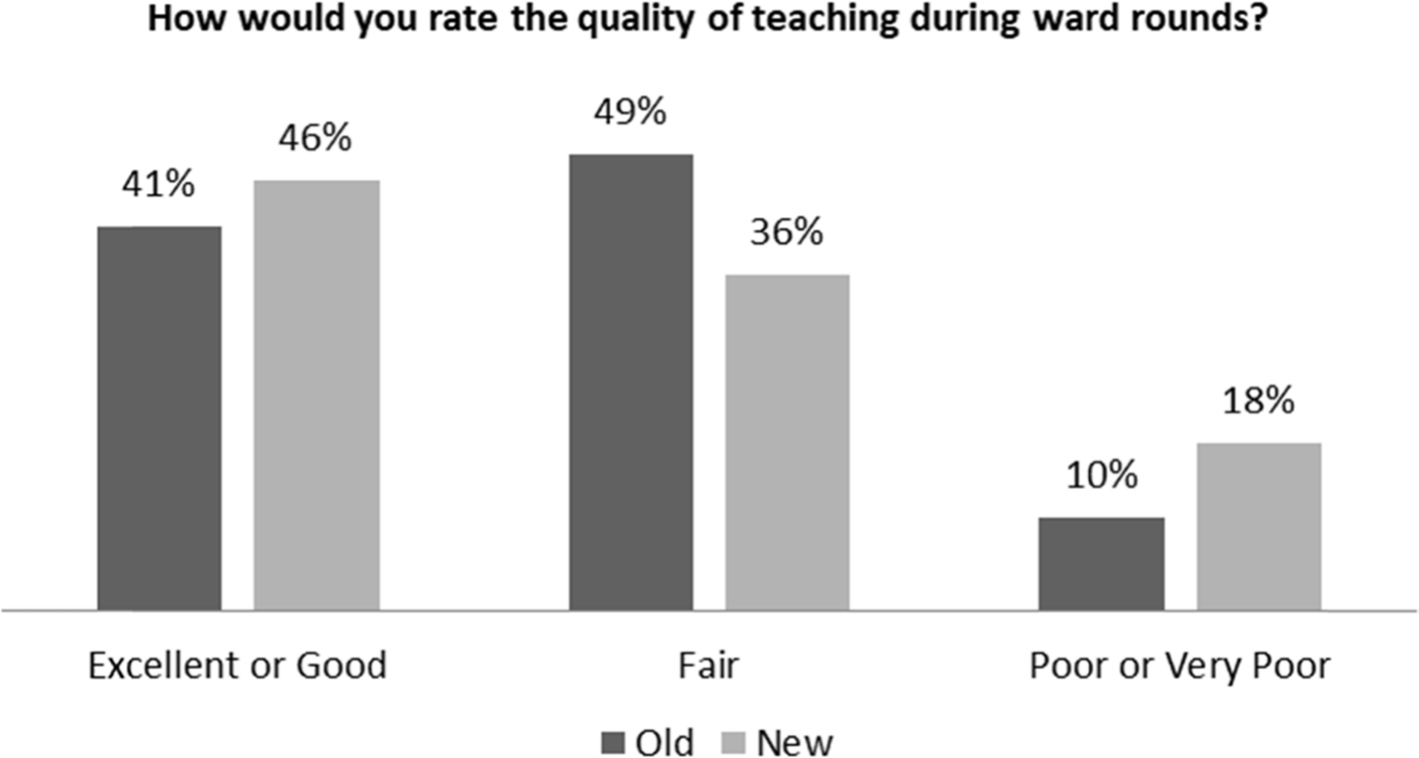
Residents’ Rating of the Quality of Teaching in the Old and New Inpatient Structures

One hundred and forty pediatric healthcare workers completed the survey (97 nurses, 39 residents, and 11 attendings). Of the 49 eligible residents who worked in the old and CTU structures, 39 (80%) completed the survey. More than 97% of the residents reported being supervised by their attending physicians daily in the CTU structure, while only approximately 15% reported the same in the old structure. With respect to the quality of teaching during ward rounds, the residents reported almost equal ratings of the quality of teaching in the old and new structures (*p* = 0.43) (Fig. 1).

**Fig. 2:**
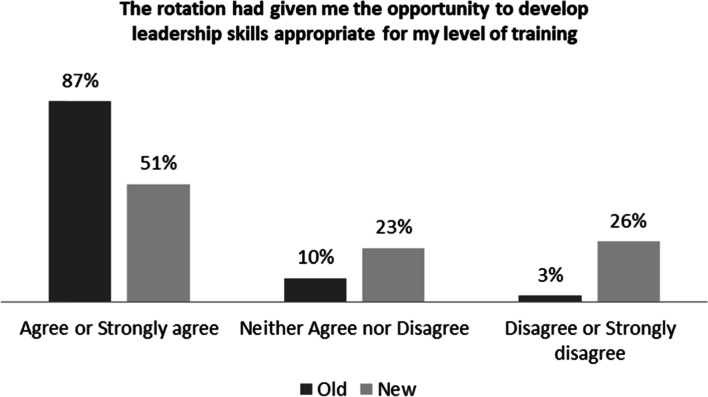
Residents Acquiring Leadership Skills in Both Inpatient Structures

When asked if the rotation gave them the opportunity to develop leadership skills appropriate to their level of training, most residents favored the old structure with 87% of them agreeing or strongly agreeing with it (*p* = 0.002) (Fig. 2).

**Fig. 3:**
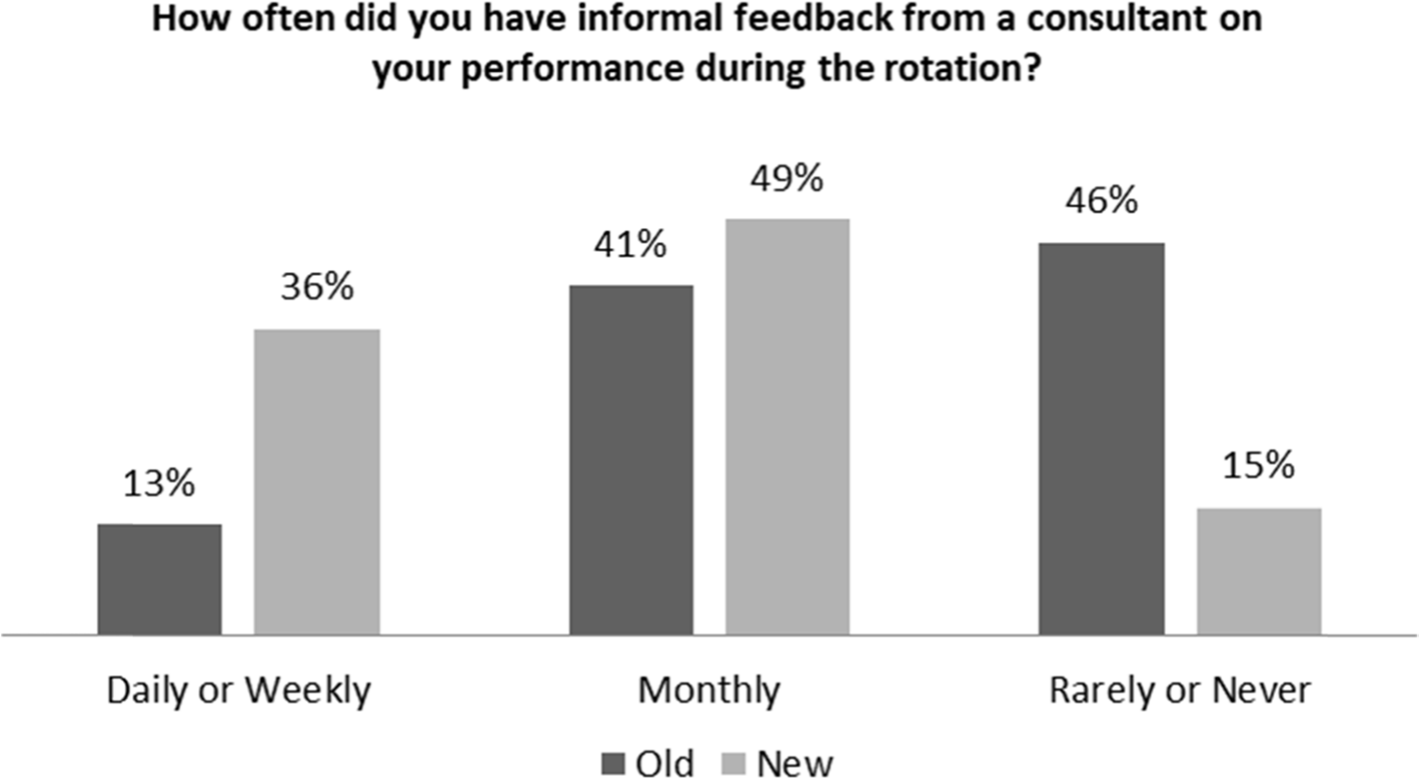
Performance Feedback in the Old and New Inpatient Structure

In the new structure, 36% of the residents reported receiving informal feedback daily or weekly, whereas 46% of them reported rarely or never receiving feedback in the old structure (*p* = 0.005) (Fig. 3).

**Fig. 4:**
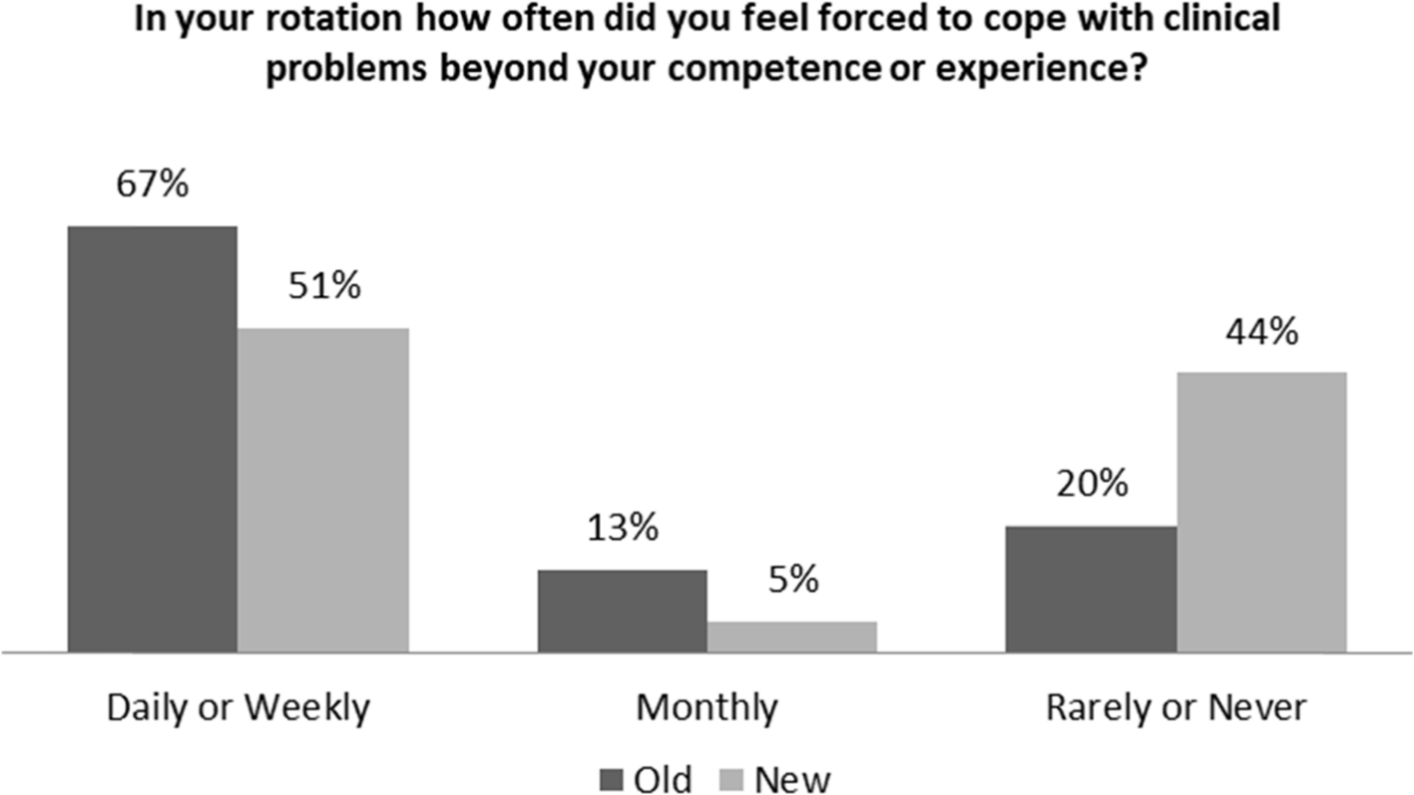
Residents’ Coping with Clinical Problems beyond Their Experience in the Old and New Inpatient Structures

Of the residents, 67% also reported that in the old inpatient structure, they were forced to cope with clinical problems beyond their area of competence or experience on a daily or weekly basis, while 41% reported that this rarely or never occurred in the new structure (*p* = 0.07) (Fig. 4).

Overall, 82% of the residents favored the CTU structure as compared to the old structure. In terms of qualitative comments, the following comments summarize the common themes perceived as positive and negative aspects of the CTU:

“think CTU structure is good, especially for the junior resident but … for the senior not always, some consultants give space to the senior to run the round and moderate discussion while other consultants do everything, which will affect the senior ability to lead and make decision”.

“Although I can see major differences between the old system and CTU, but variation between consultants is to be taken into consideration. As some of them will not give the chance to lead as senior resident and others won't attend the round at all most of the days. And you have to take decisions and relay your plan to MRP.”

“CTU system allow better patient transfer care and handover in the weekend.”

“CTU is better in different aspects but as senior I need more control over the situation as I can do the round for the old patients without consultant for more confidence”

Among the 97 nurses surveyed, 84 (87%) had worked in both the pre-CTU and CTU structures, and were included in the data analysis. Eighty percent of the nurses believed that the CTU had improved the patient safety of pediatric inpatients, 19% felt it had no effect, and 1% felt that patient safety had worsened with the new structure. Regarding the empowerment of nurses in the CTU structure, 84% of the nurses agreed that the CTU empowered them more to contact the most responsible physician (i.e., the attending) directly about their patients. Ninety-one percent of the nurses felt that overall, the CTU structure was better than the old inpatient team structure. Some selected comments from the nursing staff are as follows:

“Hoping that it will be implemented as well to other pediatric department like for example surgery.”

“It is better to have our own CTU Team in Unit 69” (Unit 69 is the high dependency unit).

“The change of Consultant every 1-2 weeks somehow affected the management of the patient in either ways…”

“I hope we also have a covering CTU physician in unit 69. We are really struggling for our own team. Thank you!”“We would like to recommend if we can have our own CTU in the unit for faster care and immediate attendance for the need of the patient for improvement of Quality of care”

Eleven pediatric consultants completed the attendings’ survey (100% response rate). The attendings rated the quality of teaching in the new structure to be either excellent (9%) or good (73%). When asked to evaluate the CTU in terms of the leadership opportunities it provides to residents that are appropriate for their level of training, 64% agreed or strongly agreed, 27% disagreed, and 9% neither agreed nor disagreed. Regarding their overall evaluation of the new structure, all attendings believed that the CTU structure was better than the old structure.

## Discussion

We present the first study in the Kingdom and gulf region to describe pediatric residents and staff perceptions of the CTU in pediatrics. Our main findings were that most residents and staff prefer the new inpatient team structure (the CTU) over the old structure especially in terms of perceived improvement in patient safety. Some residents were concerned of the lack of leadership opportunities with the CTU structure.

With daily rounds by attendings, it was not surprising that greater than 97% of the residents reported a more direct supervision in the CTU structure. This is consistent with the findings reported by Harmoen et al. (2020) that emphasized the uniqueness of the CTU structure in promoting a rich educational environment for trainees and improving clinical care through close supervision by attending consultants [7]. The quality of teaching by residents was rated almost the same in both inpatient team structures. This was somewhat surprising, as we expected the educational experience to be superior in the new structure with daily rounds by attendings. When we further analyzed our data, we found that the junior residents overwhelmingly believed that the new structure facilitated better teaching, likely due to the more direct contact with the attendings. The senior residents on the other hand were divided in their opinion as to which structure had better teaching. This needs to be examined further and may be due their perception that the teaching provided in the CTU was geared more for junior trainees.

With respect to the leadership opportunities in the two inpatient team structures, we found a significant difference in residents perceptions of the two inpatient team models. Most residents, at all levels, believed that the old structure provided them with more opportunities to develop their leadership skills. We believe that this challenge of promoting the leadership skills of residents is aligned with the already existing complexity of implementing a safe “autonomy” in the field of medical education. Autonomy is defined as the ability of a resident to manage patients on their own [8]. Autonomy in residency education is needed to develop independent and competent physicians [9]. While some residents show a clear readiness to take a leadership role in running the clinical rounds, others might feel uncomfortable to practice this opportunity while being closely supervised by their attendings. We believe in the “scaffolding model” concept as described by Hoffman, where “the role of teachers is to support the learner’s development and to provide support structures to help the learner get to the next stage of entrustment and competence”. This will help optimize the balance between autonomy and supervision [10]. In the old inpatient structure, the model allowed residents more autonomy to run rounds, especially on old patients. While this may have helped develop their leadership skills, consultant input especially on complex cases and long-term patients was often lacking. We believe this may have affected patient care and safety. With the CTU, one attending conducts daily rounds with the resident team; thus residents are supervised more closely and ultimately the quality of patient care is improved. However, if the attending is “running the rounds”, residents may not develop the required leadership skills. This was clearly expressed in the residents’ comments. Through regular meetings with residents and faculty after starting the CTU, we have strived to improve the acquisition of leadership skills during the CTU rotations. Specifically, although the attending is present on clinical rounds, senior residents are given autonomy to lead rounds and discussion with patients and their families.

Residents perceived that they received informal feedback more often in the CTU than in the old system. This is a clear benefit of the CTU model as performance feedback is key in the residents’ learning process [11]. Furthermore, in the CTU, fewer residents were forced to cope with clinical challenges or problems beyond their competencies and experiences. This is most likely the direct impact of dividing of admissions between the 4 CTU teams which resulted in the evening out and smoothing of the admission process. We believe that the more contact with attendings, timely feedback, and perceived improvement in patient safety were the reasons that most residents (over 80%) favored the CTU structure over the old inpatient structure.

Most nurses believed that the CTU had improved the patient safety of inpatients and empowered them more to discuss patient care issues with the most responsible physician (the attending). These findings were expected as the CTU involves daily rounds by the attendings. In addition, in the CTU structure, nurses were more engaged in ward rounds owing to the nature of one attending/one resident team rounding on all patients. The overall nursing staff perceptions of the CTU were so positive that the nurses recommended the implementation of this structure in other wards/services.

In this study, the attending physicians viewed the CTU as a positive change from the old structure. They rated the quality of teaching to be excellent or good. However, the attendings had some similar concerns as the residents regarding the acquisition of leadership skills during the CTU rotation.

The study has several strengths. This is the first study in the Kingdom and gulf region to describe residents and staff perceptions of the CTU structure in pediatrics. Another strength is that this study involved a heterogeneous sample of participants including residents, their attendings and nurses working in the CTU. Therefore, in this study, we could evaluate several educational and clinical/quality considerations within the CTU structure. The main limitation of this study is that it was conducted in a single center; thus the findings are difficult to generalize. In addition, this study did not assess other aspects of the CTU, including its impact on medical student/intern education.

## Conclusions

In conclusion, most pediatric residents and staff felt that the implementation of the CTU improved patient safety. The overall educational experience of residents with the CTU was better than that with the old structure, although the leadership opportunities may have decreased with daily rounds with attendings. Larger multicenter studies that involve multiple pediatrics residency programs of different sizes are needed to understand the impact of the CTU on clinical and educational outcomes.

## Supplementary Information

**Additional file 1.** Pediatric Resident Questionnaire.

## Data Availability

The datasets generated and analyzed during the current study are not publicly available due to limitations of ethical approval involving the participant data and anonymity but are available from the corresponding author on reasonable request.

## Abbreviation

CTU: Clinical teaching unit
