# Supplementary material for: Residents and staff perceptions of a pediatric clinical teaching unit in a large tertiary care center in Saudi Arabia
Source: BMC Med Educ. 2022 Feb 8;22:86. doi: 10.1186/s12909-022-03155-7 (PMC8826672; doi:10.1186/s12909-022-03155-7)
Supplement: Supplementary file 1 — Additional file 1. Pediatric Resident Questionnaire. [file 12909_2022_3155_MOESM1_ESM.docx]

**Pediatric Resident Questionnaire**

**What is your level of training?**

1. **R2**
2. **R3**
3. **R4**

**Have you had general pediatric rotations in both the old inpatient team structure and the new structure (CTU)?**

**a. Yes**

**b. No**

**(if no, the survey ends)**

**Please answer the following questions regarding your rotations in the Old General Pediatric Inpatient Structure and the New General Pediatric Inpatient Structure (CTU):**

**OLD NEW (CTU)**

1. In your rotation how often were you Daily | Weekly | Monthly | Rarely | Never Daily | Weekly | Monthly | Rarely | Never

supervised by a consultant?

1. In your rotation how often did you feel Daily | Weekly |Monthly | Rarely | Never Daily | Weekly |Monthly | Rarely | Never

forced to cope with clinical problems

beyond your competence or experience?

1. How would you rate the quality of clinical Excellent | Good | Fair | Poor | Very poor Excellent | Good | Fair | Poor | Very poor

supervision in your rotation?

1. How would you rate the quality of experience Excellent | Good | Fair | Poor | Very poor Excellent | Good | Fair | Poor | Very poor

in your rotation?

1. How would you rate the quality of teaching Excellent | Good | Fair | Poor | Very poor Excellent | Good | Fair | Poor | Very poor

during the rounds.

**OLD NEW (CTU)**

1. How confident are you that the rotation Very confident | Fairly confident | Neutral | Not very confident | Not at all confident Very confident | Fairly confident | Neutral | Not very confident | Not at all confident

will help you acquire the competencies

you needed at that particular stage of

your training?

1. Were you able to attend departmental Yes, every time | Yes, most of the time | Yes, some of the time | No Yes, every time | Yes, most of the time | Yes, some of the time | No

teaching during your rotations?

1. Handover arrangements in the rotation Strongly agree | Agree |Neither agree nor disagree |Disagree | Strongly disagree Strongly agree | Agree |Neither agree nor disagree |Disagree | Strongly disagree

between shifts ensure continuity of care

for patients.

1. How often did you have informal Daily | Weekly |Monthly | Rarely |Never Daily | Weekly |Monthly | Rarely |Never

feedback from a consultant on your

performance during the rotation?

1. The rotation had given me the Strongly agree | Agree |Neither agree nor disagree |Disagree | Strongly disagree Strongly agree | Agree |Neither agree nor disagree |Disagree | Strongly disagree

opportunity to develop leadership

skills appropriate for my level of training.

1. How would you rate the intensity of Very light | Light |About right |Heavy |Very heavy Very light | Light |About right |Heavy |Very heavy

your work by day, in the rotation?

1. In this rotation, how often have you Daily |Weekly |Monthly |Rarely |Never Daily |Weekly |Monthly |Rarely |Never

worked beyond your duty hours?

**Please answer the following 2 questions to complete the survey:**

1. Overall, which inpatient team structure do you think is better?

a. The new general pediatric inpatient structure (CTU)

b. The old general pediatric inpatient structure

1. If you have any other comments about the change in the general pediatric inpatient service to the CTU structure or how it could be improved, please enter it here:

___________________________________________________________________________________________________________________________________________________________________________________________________________________________________________________________________________________________________________________________________________________________________________________________________
